# Exploiting Microfluidics for Extracellular Vesicle Isolation and Characterization: Potential Use for Standardized Embryo Quality Assessment

**DOI:** 10.3389/fvets.2020.620809

**Published:** 2021-01-05

**Authors:** Bahram Talebjedi, Nishat Tasnim, Mina Hoorfar, Gabriela F. Mastromonaco, Marcia De Almeida Monteiro Melo Ferraz

**Affiliations:** ^1^School of Engineering, University of British Columbia, Kelowna, BC, Canada; ^2^Reproductive Sciences, Toronto Zoo, Toronto, ON, Canada; ^3^Department of Veterinary Sciences, Ludwig-Maximilians-Universität München, Munich, Germany

**Keywords:** embryo, extracellular vesicles, microfluidics, embryo selection, extracellular vesicles isolation

## Abstract

Recent decades have seen a growing interest in the study of extracellular vesicles (EVs), driven by their role in cellular communication, and potential as biomarkers of health and disease. Although it is known that embryos secrete EVs, studies on the importance of embryonic EVs are still very limited. This limitation is due mainly to small sample volumes, with low EV concentrations available for analysis, and to laborious, costly and time-consuming procedures for isolating and evaluating EVs. In this respect, microfluidics technologies represent a promising avenue for optimizing the isolation and characterization of embryonic EVs. Despite significant improvements in microfluidics for EV isolation and characterization, the use of EVs as markers of embryo quality has been held back by two key challenges: (1) the lack of specific biomarkers of embryo quality, and (2) the limited number of studies evaluating the content of embryonic EVs across embryos with varying developmental competence. Our core aim in this review is to identify the critical challenges of EV isolation and to provide seeds for future studies to implement the profiling of embryonic EVs as a diagnostic test for embryo selection. We first summarize the conventional methods for isolating EVs and contrast these with the most promising microfluidics methods. We then discuss current knowledge of embryonic EVs and their potential role as biomarkers of embryo quality. Finally, we identify key ways in which microfluidics technologies could allow researchers to overcome the challenges of embryonic EV isolation and be used as a fast, user-friendly tool for non-invasive embryo selection.

## Introduction

The selection of the most fit embryo (i.e., highest developmental potential to result in a pregnancy and birth of a healthy offspring) for transfer after *in vitro* production remains one of the biggest challenges faced by embryologists today. Historically, evaluation of embryo quality has been performed by analyzing embryo morphology under a light microscope (1, 2). It is becoming clear, however, that embryo competence can be compromised in morphologically normal embryos (3), as evidenced by differences in *in vitro* and *in vivo* embryo transcriptomics and methylomics (4, 5), which could result in implantation failure or miscarriages early in pregnancy (6, 7). Invasive pre-implantation genomic testing for aneuploidy has improved embryo selection since the transfer of chromosomally normal embryos has resulted in better implantation rates and reduced miscarriages (7, 8). However, ~30% of transferred, chromosomally normal embryos still fail to implant, demonstrating a clear need to improve embryo selection prior to transfer (9). In recent years, the evaluation of embryo morphokinetics has also been used, and has been positively correlated with implantation success (10, 11). Moreover, embryo metabolism has also emerged as a method for evaluating embryonic competence. The assessment of specific metabolites, such as oxygen, amino acids, glucose, lactate, leptin, and sHLA-G, were already described as possible markers for embryo selection (12–14). Although, current studies have provided a glimpse of the diverse metabolic mechanisms used by pre-implantation embryos, we have only scratched the surface in understanding these mechanisms and their control (15). There lacks a clear understanding of the broader context of the embryonic metabolic environment, and importantly, of which pathways to use to promote optimal quality (15).

**Figure 1:**
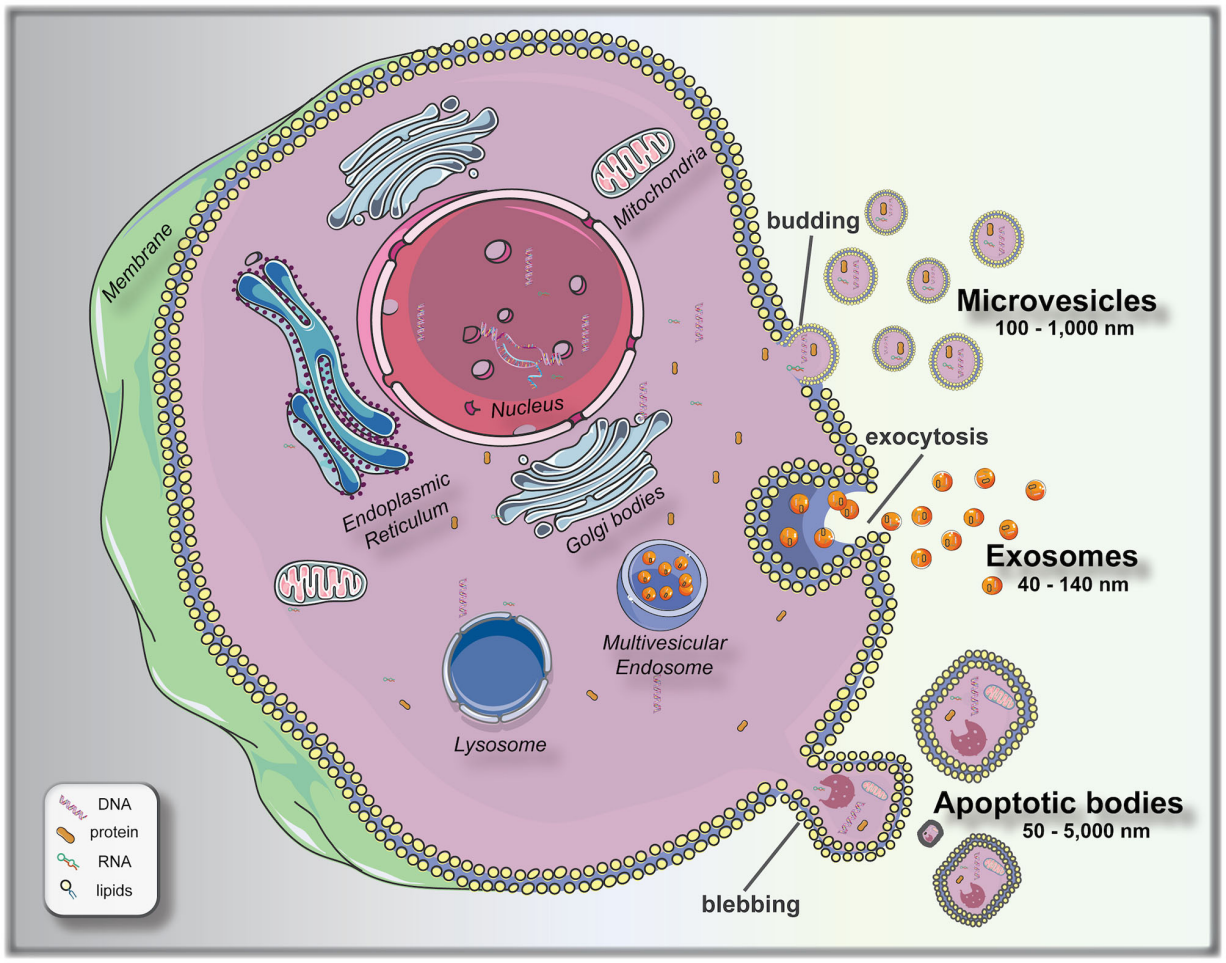
Biogenesis and release of extracellular vesicles (EVs). Microvesicles bud directly from the plasma membrane; exosomes are formed by budding of small vesicles into early endosomes and multivesicular endosomes, which are released by exocytosis; and apoptotic bodies are released by outward blebbing of apoptotic cells (16). This figure was created using Servier Medical Art templates, which are licensed under a Creative Commons Attribution 3.0 Unported License (https://smart.servier.com).

To bridge this knowledge gap, significant attempts are being made at finding measurable and reproducible non-invasive variables to be used as biomarkers for pre-implantation embryo selection. Following this principle of non-invasive biomarker search, extracellular vesicles (EVs) arise as a promising field to elucidate different aspects of embryo biology. EVs are membrane encapsulated units carrying regulatory molecules, including proteins, peptides, RNA species, lipids, and DNA, that have emerged as an important mechanism of cell communication (16, 17). EVs are classified as: exosomes (40–140 nm in size), secreted from most cell types after fusion of a multivesicle body with the plasma membrane (exocytosis); microvesicles (100–1,000 nm), shed via budding from the plasma membrane; and apoptotic bodies (50–5,000 nm), which are outward budding or blebbing from apoptotic cells (18, 19) (Figure 1). The release and uptake of EVs by cells depend on different stimuli, cell signals and biochemical stressors (9). Consequently, EVs have been isolated from most bodily fluids, and it is evident that they have a key role not only in the regulation of physiological processes, but in the pathology underlying several diseases. Different studies have been performed to elucidate the versatile roles of mammalian cell-released EVs in health and disease, and their use as biomarkers for diseases, such as prostate cancer (20), pre-eclampsia (21), breast cancer (22), glioblastoma (23), neurological disease (24), among others.

Although significant effort has been placed onto this nascent field of research, the use of EVs as biomarkers of embryo quality remains limited by the lack of exclusive biomarkers, limited sample volumes and concentrations, inefficient separation methods, and most importantly, the lack of an automated, user-friendly, reproducible method for isolating and characterizing EVs in low concentration, small-volume samples. In this regard, microfluidics has emerged as a promising tool to create platforms for processing small amounts of fluids (microliter to picoliter) (25, 26). Besides the small-volume capacity, microfluidics protocols can be performed with a high level of purity and sensitivity, while reducing the cost, the volume of reagents expended, and time invested in the process (27, 28). In this review, we aim to summarize the knowledge of the most commonly used methods for EV isolation, elaborate on the possibilities offered by microfluidics to improve and standardize current EVs isolation methods, and provide insight on the use of EVs as biomarkers of pre-implantation embryo quality. Finally, we debate the prospective applications of integrated microfluidic platforms for isolating and characterizing EVs from embryo culture media, for embryo quality analysis and their potential use as diagnostic devices in the near future.

## Conventional EV Isolation Methods

Conventional methods for isolating and purifying EVs from a broad range of biofluids are used widely in established laboratory and clinical protocols. These methods isolate EVs on the basis of their physical properties, such as size and density (ultracentrifugation, density gradient, filtration), and physicochemical interactions such as their differential solubility (chemical precipitation), and protein expression (affinity capture) (29). These techniques can be classified into four subgroups based on: (1) ultracentrifugation (UC), (2) filtration (3) precipitation and (4) affinity (Table 1). All conventional techniques vary in their specificity and recovery (i.e., yield) (30). According to the Minimal Information for EV Studies guidelines (MISEV2018), the yield of EV isolation methods can be positioned within a recovery vs. specificity grid that ranges from low to high along each dimension (31). Concentration or “enrichment” of EVs is performed with or without separation using techniques such as UC and precipitation, which increase the EV count per unit volume or relative to another component. Separation of different types of EVs from each other is more challenging and can be achieved to various degrees by immune- or other affinity isolation. Some complementary techniques can be combined into multi-step isolation protocols as opposed to single-step procedures, depending on the downstream analysis (33). The choice of isolation protocol is made based on the type of biofluid, nature of research (e.g., basic vs. clinical), experimental question, scalability and reproducibility of the technique, and EVs end-use (32). As a result, isolation approaches can vary between studies, depending on the downstream application and scientific question. Though some effort has been made to standardize the EV isolation methodologies and create best-practice guidelines (e.g., EV-TRACK) (34), the lack of community standards and the inability of current methods to isolate EVs with high enough quality and quantity have created a major “isolation problem” in the field of EV research (35, 36). Highly purified EVs are needed to attribute a function or a biomarker to vesicles as compared with other excretory-secretory bioparticles, especially when evaluating the relative importance of EVs for a detected function. Absolute purification of EVs from non-vesicular substrates (i.e., with high recovery and specificity) remains a major challenge for common EV isolation protocols. Contaminants such as free proteins, ribonucleoproteins, and lipoproteins may co-isolate with EVs and impact the yield, diversity and functions of recovered EVs (37, 38). Therefore, conventional isolation methods have intrinsic limitations that impair their use in biomarker discovery. The following sections describe these established methods, their working principles, and their advantages and shortcomings.

**Table 1:** Comparison of conventional EV isolation methods [based on (30–32)].

**Isolationmethod**	**Sample volume**	**EV size(nm; on average)**	**Recovery**	**Specificity**	**Processing time (h)**	**Major contaminant**	**Major artifact**
Ultracentrifugation based methods	μL–mL	20–100	Low to intermediate	Low to intermediate	2–96	Similar sized contaminants and lipoproteins	EV-particle aggregates
Size based methods	μL–mL	50–200	Intermediate	Intermediate	0.3–2	Same size particles	EV-particle aggregates
Precipitation based methods	μL–mL	60–180	High	Low	0.3–12	Protein	Protein complex, EV-particle aggregates
Affinity based methods	μL	40–150	Low	High	4–20	Soluble proteins	

“*Recovery” refers to the percentage of total EVs preserved after isolation. “Specificity” refers to the recovery of a subtype (or few subtypes) of EVs*.

### Ultracentrifugation Based Isolation

Ultracentrifugation is currently considered the gold standard for EV isolation from different biofluids, and the technique of choice in approximately 56% of all laboratories (39). Centrifugation is a single-step and label-free method that isolates EVs by accelerating their natural sedimentation rate, which is based on the difference of their density compared to the surrounding media. The protocol consists of successive centrifugation steps with increasing centrifugal force (g) to remove contaminants that are larger than the EVs and then pellet the EVs. The efficiency of EV isolation by centrifugation depends on many factors, such as acceleration force, type of rotor and its characteristics, and sample viscosity (30, 40). The main limitations of UC are that it is time-consuming, requires expensive ultracentrifuge equipment, and results in relatively low recovery of EVs (41, 42). Different UC techniques have been developed to isolate smaller from larger EVs selectively. These methods are discussed further in the following subsections.

#### Differential Ultracentrifugation

Differential UC was the first method used to isolate EVs (43) and continues to be the most common method to concentrate EVs from body fluids and cell media (38, 44). It involves the use of successive centrifugation steps with varying *g* forces to separate particles. In this method, a low-speed centrifugation pre-step removes cell debris, after which vesicles are isolated at 19,000–100,000 × *g* (45). This enables the enrichment or concentration of different EVs fractions; however, complete separation is not achieved. The disadvantages of differential UC are associated with the use of high centrifugal forces and density-dependent isolation. Vesicles can clump as a result of protein aggregation at high velocities (46). Because centrifugation separates particles by density, the recovered pellet contains both EVs and smaller contaminants with similar density, such as viruses, proteins, protein aggregates, lipoproteins and cellular debris. As a result, classical differential UC can result in low recovery of small EVs (5–25% of starting concentration of exosomes) (47). This technique is, therefore, more appropriate for laboratory applications than clinical; however, isolation protocols can vary between labs resulting in inconsistencies from different centrifugation time, speed, type of rotor or other technical factors (e.g., temperature) (34). Additionally, this method is not well-suited for the recovery of EVs from high viscosity biofluids such as plasma (40). Newer UC based techniques are being designed for the selective isolation of EVs subtypes by combining centrifugation with ultrafiltration or size-exclusion chromatography (45) (see section Filtration Based Methods).

#### Density Gradient/Cushion Ultracentrifugation

Density-gradient ultracentrifugation (DGUC) is a variation of the UC method that includes an additional sucrose gradient/cushion step or OptiPrep™ velocity gradient to separate EVs based on their buoyant densities with increased sample purity. In this method, a sucrose gradient (20–60%)/cushion (30% sucrose) or 5–40% iodixanol gradient is incorporated into the centrifugation protocol to purify and isolate specific EV fractions (density range between 1.13 and 1.19 g/mL) and eliminate contamination (48). Once EV-rich fractions are obtained, they are usually purified with UC or size-exclusion chromatography (see section Ultrafiltration). Sucrose is the most commonly applied density gradient for EVs isolation; however, sucrose-DGUC protocols may result in the loss of biological function due to the applied gradient being hyperosmotic and the extreme g-forces leading to disruption and loss of EVs (30). Unlike sucrose, iodixanol-DGUC forms iso-osmotic solutions at all densities and preserves the sizes of EVs in the gradient (49). The DGUC approach is commonly a two-step isolation method, involving direct UC followed by DGUC, but new single-step methods are being developed (50). Although DGUC allows higher purity separation of EVs based on their densities, some high-density lipoproteins (HDLs) can be co-isolated (51). This method also suffers from the impracticalities associated with UC (i.e., long turnaround time, specialized equipment).

### Filtration Based Methods

Numerous protocols for EVs isolation are based on separating them by their size using filtration methods, which are discussed in the following subsections.

#### Ultrafiltration

Ultrafiltration (UF) allows the separation of EVs from soluble components. The soluble components are eluted using a filter and either applying pressure or placing in an ultracentrifuge. Because of the applied pressure, deformable EVs may pass through the filter. UF is more time efficient than UC and is effective at concentrating EVs, requiring about 20 min to recover up to 80% EVs and concentrate them up to 240-fold (52). Many commercially available filtration methods are based on sieving the sample through a nanoporous membrane using centrifugation, pressure or vacuum; however, UF alone is not applicable for EV isolation since there are no standardized protocols and EVs can be lost through irreversible binding to the membranes or through blockage of the membrane pores by protein aggregates (30, 35).

#### Size-Exclusion Chromatography

Size-exclusion chromatography (SEC), also referred to as “gel filtration,” is a single-step liquid chromatography technique that isolates EVs based on their size distribution in solution (53). It involves eluting EVs on a single column (either commercial or homemade) based on a size cutoff determined by choice of exclusion matrix (54). SEC is the method of choice for the isolation of high concentrations of EVs from highly complex fluids such as plasma with very low co-isolation of contaminants (99% soluble plasma proteins and >95% HDLs removed) (55). For solutions such as cell culture media, which are less concentrated in EVs, the EVs are pre-concentrated with UF (UF-SEC) (56). The SEC approach has the advantages of recovering EVs with more intact biophysical composition, smaller size distribution profile, and higher functionality (57–59). The sample processing time is short (20 min), and the SEC components are inexpensive, although sample preparation and washing are still relatively laborious. One of the main disadvantages of using liquid chromatography techniques is that manual collection is required, which may introduce operator-related variability, and make comparisons across samples challenging to impossible. Another limitation is the dilution of the purified sample, which requires additional concentrating steps and may reduce recovery/yield. Compared to UC, there is no risk of protein complex formation and vesicle aggregation or contamination with particles having overlapping densities (53), however, SEC only permits efficient isolation of EVs larger than the pore size of the column matrix (55). The column height, column diameter, pore size of matrix and sample volume can be optimized to improve the recovery and purity of isolation. Newer commercially available core bead chromatography techniques using bind-elute SEC (BE-SEC or BEC) columns have been shown to purify EVs time efficiently with 80% recovery (60). The BEC method is multistep and requires samples to be pre-concentrated with spin filters (usually 100 kDa) using Tangential Flow Filtration (TFF) to reduce impurities from being loaded into the column. This combined TFF-BEC can process larger volumes of conditioned media (up to 200 mL) (60).

### Precipitation Based Methods

Precipitation based methods are the second most popular method after UC (used in 26% of all research papers) (30). Precipitation kits/polymers are based on the fact that EVs change their solubility and/or aggregate, instead of isolating them based on their density and size. The most widely used precipitation method is discussed further in the following subsection.

#### Polymeric Precipitation

Polymeric precipitation is based on the formation of a mesh-like polymeric web that captures EVs of a smaller size range, usually between 60 and 180 nm, which are later pelleted at low centrifugal speeds. Multiple commercial precipitation kits are available to precipitate EVs from different biofluids. These kits are based on the super hydrophilic volume-excluding polymer poly-ethylene glycol (PEG) to isolate EVs based on their decreased solubility in PEG solutions (30). PEG is a non-toxic and non-denaturing water-soluble synthetic polymer and is the most effective in terms of both its precipitating ability and cost. In most methods, samples are incubated with PEG at 4°C for up to 12 h and centrifuged at low speed to collect precipitated EVs in the buffer. Since EVs are negatively charged due to the presence of phosphatidylserine on their surface, charge-based precipitation could also be performed in the presence of PEG by using positively charged molecules such as protamine (61). PEG has also been used along with dextran to create a two-phase system for isolation that significantly decreased protein contamination (62). Conversely to PEG, some precipitation methods rely on hydrophobic interactions to aggregate EVs by “salting them out” using sodium acetate; however, this procedure is non-specific (63). Precipitation allows for a greater yield of the EVs (90% recovery); however, it can also co-isolate lipoprotein contaminants, especially from serum samples (42, 52). It should also be noted that although this method is inexpensive, requires no special equipment and is comparable between low and high sample volumes, PEG precipitation is not suitable for EV biomarker identification since it concentrates EVs (32). For biomarker identification, EVs should be isolated before concentration by precipitation.

### Affinity Based Methods

Affinity based methods are a new and upcoming solution to the EV isolation problem. Their working principle is based on adsorption or ionic interactions with the molecules on the EV outer surface. Although these methods are multi-step and time-consuming, they can isolate specific EV subtypes with high recovery. Different affinity-based isolation techniques are discussed in the subsequent sections.

#### Immunoaffinity Capture

Immunoaffinity purification or immunoprecipitation (IP), also known as immuno-capture, exploits the presence of characteristic surface proteins on certain EV classes. Specifically, this method relies on the use of antibodies to target EV receptors/surface markers such as tetraspanins (CD9, CD63, and CD81), heat shock proteins and MHC antigens (64). Antibodies can be used to select EVs either positively (immune-enrichment) or negatively (immune-depletion) from culture media or body fluids (35). IP assays trap/adsorb EVs using a diversity of antibodies coated on fixed phases such as magnetic/non-magnetic microbeads, silica microtips, surface plastic plates, cellulose filters or membrane affinity filters, and many proprietary IP based isolation kits are commercially available [reviewed in (30)]. IP techniques are more efficient and economical than commercially available precipitation or sedimentation-based EVs purification kits (65). IP protocols can isolate EVs subpopulations with high specificity regardless of size (62); however, this concomitantly lowers recovery/yield compared to methods that rely on EVs physical properties (48). Other affinity capture methods have been developed that target proteoglycans, heat shock proteins, phosphatidylserine, glycoproteins and glycolipids, however, many of these approaches require pre-isolation via methods such as UF and are therefore more complex and time-consuming (30, 66).

#### Affinity and Ion-Exchange Chromatography

Both affinity chromatography (AC) and ion/anion-exchange chromatography (AIEX) are affinity-based chromatographic EV isolation techniques with the advantage of higher throughput. AC is based on interactions with immobilized ligands on the stationary phase, while AIEX uses a positively charged matrix to attract negatively charged EVs (54). The AC strategy involves tagging EVs for capture in the affinity columns, which can then be eluted to recover enriched tag-specific EVs (67). The AIEX protocol has been recently improved to enable the isolation from up to 1 L of cell media within 3 h with fewer protein contaminants than UC and TFF (68) and sucrose- DGUC (69).

## Microfluidics for EV Isolation

**Figure 2:**
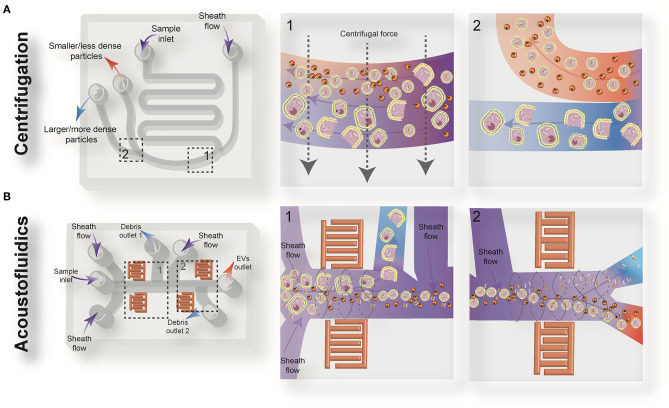
Schematic view of the active microfluidic-based methods for extracellular vesicles (EVs) isolation: on-chip centrifugation **(A)** and acoustofluidics **(B)**.

**Figure 3:**
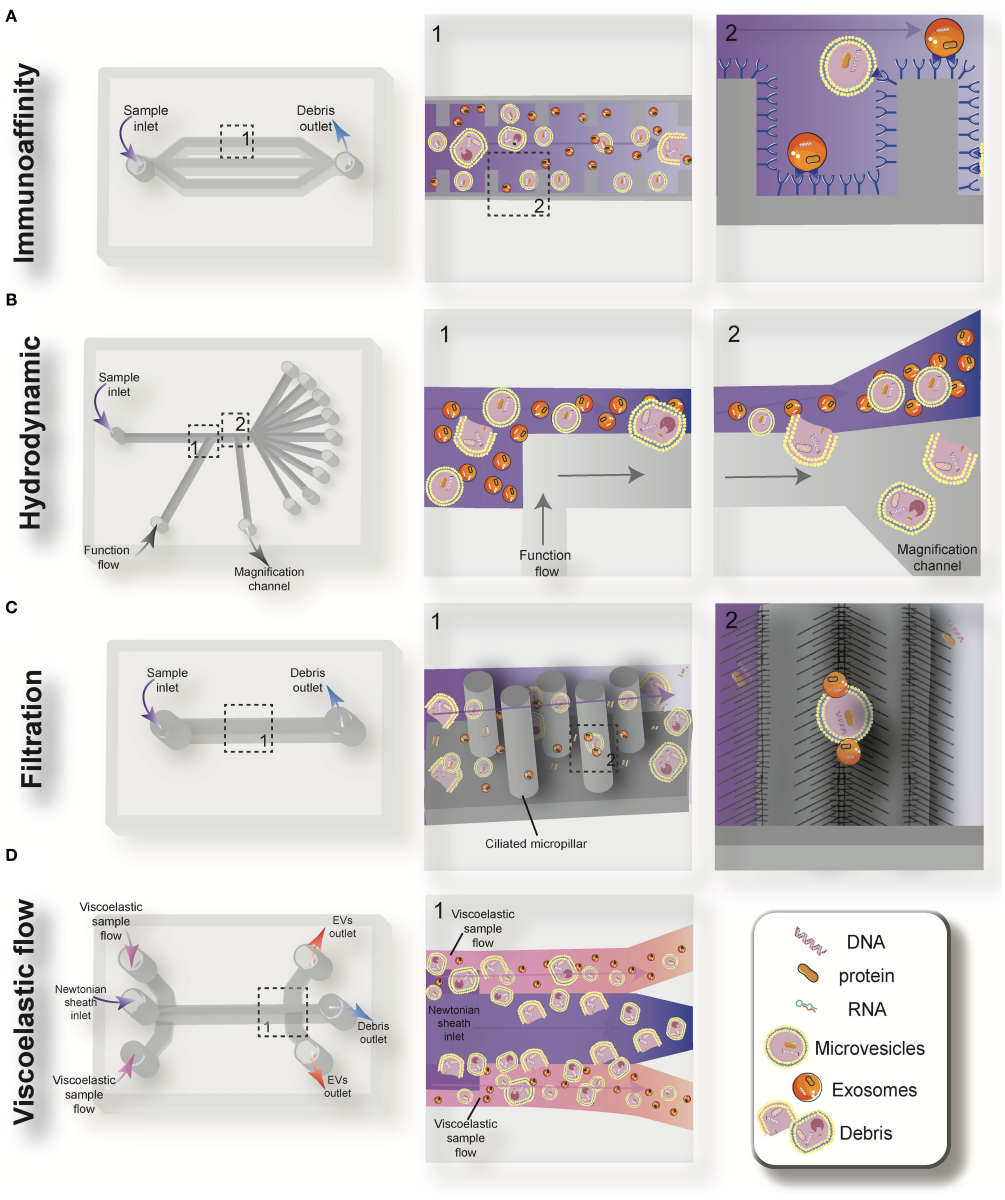
Schematic view of the passive microfluidic-based methods for extracellular vesicles (EVs) isolation: immunoaffinity **(A)**, hydrodynamic **(B)**, filtration **(C)**, and viscoelastic flow **(D)**.

Driven by recent advances in microfabrication science, microfluidics has emerged as a low-cost and promising tool for biological studies. The highly efficient separation and isolation of micron or nano-sized particles inside a particular volume of fluid are among the remarkable advantages of microsystem devices. This attracts research attention for the on-chip assessment of biological samples. It has been shown that microfluidic techniques enable an accurate and sensitive sorting of different subpopulations of EVs by reducing the consumed reagents, manufacturing cost, and experimental time (70–72). Aside from immunoaffinity-based EV isolation methods, microfluidics offer label-free extraction of EVs, which only rely on the physical properties of the EVs, regardless of their biochemical properties. Size or density-based separation techniques are the most frequently used label-free methods on both active and passive microfluidic platforms. Although active microdevices consist of multiple complex components and require advanced equipment for the manufacturing process, they reveal better performance for nano-scaled size separation and can be rapidly adjusted for a specific desired size. Here, different microfluidics-based active (centrifugal and acoustic microfluidic; Figure 2) and passive techniques (immunoaffinity, filtration, viscoelastic, and hydrodynamic flows; Figure 3) for EV isolation will be elaborated and discussed. These techniques are summarized in Table 2.

**Table 2:** Comparison of microfluidics-based EV isolation methods.

**Isolation approach**	**Sample**	**Sample volume**	**EV size (nm)**	**Recovery**	**Specificity**	**Processing time**	**References**
On-chip centrifugation	Cell culture media	<10 μL	20–1,000	Intermediate to high	Low to intermediate	<4 min	(73)
Acoustofluidic separation	Whole blood	<300 μL	30–1,000	High	Intermediate to high	<30 min	(71, 74)
Filtration	Urine	<100 μL	20–6,200	Intermediate to high	Intermediate to high	<10 min	(75, 76)
Viscoelastic flow	Serum, cell	<100 μL	30–200	High	Low to intermediate	<5 min	(77)
Hydrodynamic mechanism	Urine	<500 μL	20–110	Intermediate to high	Low to intermediate	3–7 min	(78)
Immunoaffinity capture	Serum	20–100 μL	30–300	Low to intermediate	High	20–40 min	(79–81)

“*Recovery” refers to the percentage of total EVs preserved after isolation. “Specificity” refers to the recovery of a subtype (or few subtypes) of EVs*.

### On-Chip Centrifugation

Centrifugal microfluidic platforms have been applied to different biomedical fields such as drug discovery, mixing of various reagents, plasma separation, cell lysis, analyte detection, and colorimetric detection of biomarkers (82–85). Compared to other chip-based separation techniques, centrifugal microfluidic platforms are mounted on a simple motor to impose desired forces for the liquid manipulation process, thereby eliminating the need for syringe pumps to introduce the sample into microchannels. The centrifugal “lab-on-a-disk” allows density-based separation of particles by imitating the classical sedimentation and separation techniques in an automatic manner at smaller scales (86). The density-based approaches benefit from the differential centrifugal force acting on separated elements, causing the denser objects to sediment faster in a radially outwards direction along the centrifugal force vector, while the supernatant objects are transferred downstream (87). In 2018, Yeo et al. introduced a label-free centrifugal microfluidic model for the extraction of EVs from cell culture medium (73). Their microdevice was used to separate bioparticles below 100 nm in a few minutes with 90% efficiency and 85% purity. The device consisted of three main sections: the microfluidic chip, the rotor assembly, and the centrifugal rotor. The microfluidic chip consisted of one serpentine inlet (to provide sufficient hydrodynamic resistance toward fluid movement), a separation segment and two outlets for size-selective separation (Figure 2A). In the microdevice, various forces, including drag, Coriolis, pressure, buoyancy, and Euler forces, act on the particles changing their path to one of the outlets. The direction of the centrifugal force is perpendicular to the axis of fluid flow and is counteracted by hydrodynamic drag, buoyancy, and Coriolis forces. The resultant terminal velocity of nanoscale-sized EVs moves them toward the outer channel wall. This migration is dominated by centrifugal force, which is proportional to the square of particle diameter. As a result, larger particles experience larger centrifugal force and migrate longer distances. In this way, microvesicles can be separated from exosomes at a second outlet (73).

### Acoustofluidic Separation

In this technique, a pair of interdigital transducers (IDTs) are patterned on a piezoelectric substrate, usually lithium niobate (LiNb*O*_3_) wafer, to form an acoustic field inside the fluid domain. Because of the inverse piezoelectric effect, applying a sinusoidal signal to the IDT results in the propagation of two surface acoustic waves in the piezoelectric domain (88). The interaction of counter-propagating acoustic waves produces a standing surface acoustic wave (SSAW) field within the microchannel. The pressure fluctuation induced from SSAW leads to the generation of pressure nodes (minimum pressure amplitude) or antinodes (maximum pressure amplitude) in the medium, generating acoustic radiation and viscous forces. The magnitude of acoustic radiation force is proportional to the volume of the EVs, while the magnitude of the viscous force is proportional to the radius of EVs. Therefore, larger EVs experience more acoustic radiation force and move to the pressure nodes or anti-nodes faster than smaller EVs. Particles with positive acoustic factor (such as cells and vesicles suspended in aqueous solutions) move to the pressure nodes, while bioparticles with negative acoustic contrast factor (such as some subgroups of lipoproteins) move to the pressure anti-nodes by the acoustic radiation force. The location and the number of pressure nodes can be tuned to adjust the desired cut-off size and lateral translation of the target size of EVs along the cross-section of the channel, so EVs can be easily fractionated based on their intrinsic physical properties and isolated in the desired outlet.

Wu et al. (74) developed an on-chip acoustic-based platform for continuous separation of exosomes from an undiluted blood sample. The proposed microdevice constituted one cell-removal module and one exosome-isolation module arranged in series (Figure 2B). The first unit extracts suspended particles larger than 1 μm, such as red blood cells (RBCs), white blood cells (WBCs), and platelets (PLTs), and provides a cell-free plasma for downstream nanoscale EV separation. Exosomes are then isolated from other subgroups of EVs including microvesicles and apoptotic bodies by the second unit. The total EV isolation time for a 100 μL sample of undiluted human blood is about 25 min with 98% purity and 82% yield (74). EVs and lipoproteins are both nanoscale bioparticles and overlap in size distribution, which makes their separation extremely challenging with conventional isolation methods. To overcome this challenge, an acoustofluidic-based separation strategy for the separation of EV and lipoprotein subclasses based on the differences in acoustic properties has been created by precise implementation of a SSAW on a microchannel (88). The subgroups of lipoproteins with negative acoustic contrast factor are collected in a negative contrast outlet in the center of the microchannel, and purified EVs are focused in two sidewalls and collected in a positive contrast outlet (side outlets) (88). In this device, EVs can be separated from very low density lipoproteins, with a 70% purity.

### Immunoaffinity Capture

Immunoaffinity-based separation of EVs in microfluidic devices is accomplished through coating the microchannels with antibodies or introducing antibody-coated magnetic beads in the microchip (79, 89). These methods show the most promise for the separation of specific subtypes of exosomes from other subpopulations of EVs. Recent work has shown that microfluidic immunoisolation methods could be considered as a powerful alternative method for conventional tumor biopsy. As an example, He et al. developed a cascading microfluidic device to analyze the plasma-derived exosomes of patients with lung cancer by integrating on-chip immunomagnetic isolation with *in situ* protein analysis. They demonstrated that their exosome analysis platform possesses excellent capacity for screening cancerous from non-cancerous samples (80). In 2016, Zhao et al. introduced a microfluidics methodology (named Exosearch) for the large-scale enrichment of tumor-derived circulating exosomes by employing magnetic beads conjugated with antibody probes. Three different types of exosomal markers (CA-125, EpCAM, CD24) were used for the early diagnosis of ovarian cancer with high accuracy (81). An affinity-based microfluidic device for exosome capture from ovarian cancer serum has also been developed (79). In this device, the microchannel was fabricated with herringbone grooves functionalized with antibodies against CD9 and EpCAM (epithelial cell-specific marker) (Figure 3A). The herringbone grooves improved the capturing efficiency by increasing the fluid-surface interaction. Using this method, the captured exosomes remained intact and available for subsequent downstream analysis. Compared to conventional separation techniques, this platform had a lower volume sample requirement (100 μl), a shorter processing time (<20 min), improved yield, and greater specificity (79). One of the most promising ways to enhance the specific immunocapture of target EVs subpopulations is the incorporation of nanostructured coatings in the microfluidic devices. For instance, Zhang et al. employed a nanostructured graphene oxide/polydopamine (GO/PDA) interface with a Y-shaped arrangement of microposts to provide a larger capturing surface area for targeted EVs and improve the mixing quality. This nano-IMEX microchip enabled the ultrasensitive molecular analysis and quantitative detection of exosomes derived from patients with colon and ovarian cancer (90).

### Hydrodynamic Mechanism

Deterministic lateral displacement (DLD) is a hydrodynamic microfluidics method for the manipulation of different-sized particles by using an array of microposts (78). The pattern of microposts determines the perpendicular translation of suspended particles in relation to the direction of primary flow. The particles below the specific size follow the main stream flow with no deflection, while bigger particles experience lateral deviation and separate from the main suspension. The gap between pillars and the offset of posts are the two main factors that determine the critical cut-off size. Wunsch et al. (78), developed a nanoscale DLD methodology by means of manufacturable silicon processes method for the size-based separation of particles between 20 and 110 nm with sharp resolution. They fabricated pillars with gap sizes ranging from 25 to 235 nm, which successfully fractionated the polydisperse population of exosomes based on their size (78). Shin et al. (91) presented a microfluidics methodology for the size-based separation of EVs without hampering their functionality. Their device could sort EVs of heterogeneous sizes (0.1 and 5 μm) in less than an hour. The device consisted of two inlets for sample and function flow, one magnification channel for flow withdrawal and nine outlets (Figure 3B). The function flow aligns the nano-vesicles and micro-particles to the upper wall of the channel. After the particle alignment, the channel was expanded by 21-fold and entered into to the pinching region, which was also expanded by 21-fold, where the slight difference in the position of particles in the pinch section amplified in the broadened part, to enable the size-based separation of suspension through multiple outlets. This separation principle is similar to the pinched-flow fractionation method described in Pamme (92). To weaken the effects of Brownian motion and maintain the dominance of inertia forces on the bioparticles, flow rates are set as relatively large measures (50–200 μL/min). For providing larger lateral displacement of particles and improving the accuracy of the system, unlike conventional microfluidics systems, which work with Reynolds number (ratio of inertial forces to viscous forces within a fluid, which is subject to relative internal movement due to different fluid velocities, RN) smaller than 1, in this study, the RN for the pinched and broadened region was set in the range 27–54 and 1.8–3.5, respectively. Using this device, exosomes were collected in outlets 1–3, while bigger EVs, such as apoptotic bodies, were detected at outlets 5–9. Analysis of outlet 2 samples demonstrated exosomal cup-shaped morphology with the size range between 30 and 100 nm, whereas outlet 8 samples showed apoptotic bodies and large size particles (500–2,000 nm) (91).

### Filtration

Filtration is a powerful technique for the continuous separation and enrichment of sub-cellular vesicles without any need for active components. Liang et al. (75) developed a double-filtration microfluidics platform to separate, enrich, and quantify urinary EVs within the size range of 30–200 nm with a recovery rate of 74.2%. Two polycarbonate membranes with pore sizes of 200 and 30 nm were embedded in the microchip to fractionalize and enrich the EVs. Particles larger than 200 nm were trapped by the 200 nm pore-sized membrane in the sample chamber, while particles smaller than 30 nm passed through the second filtration membrane and were collected in the waste chamber. The recovery and specificity of the proposed device was 81.3 and 90%, respectively (75). Another microfluidic device for the isolation of exosome-like lipid vesicles from proteins and cell debris was fabricated with ciliated micropillars (93). This nanowire-on-micropillar structure was formed by metal assisted chemical etching, with a depth of 400 nm (94). The porous silicon nanowires on the side walls of the micropillars trapped objects in the exosome size range while other molecules passed without capture (Figure 3C). The selectivity and functionality of the device could be increased by preloading the 6–10 nm pores, at the porous silicon nanowires, with antibodies (93). Woo et al. (76) introduced a sensitive method of EV isolation by integrating centrifuge microfluidics with two nano-filters called Exodisc. Exodisc allowed the enrichment of EVs (20–600 nm) in only 30 min, and with a high recovery rate (95%). In this device, the disc was spun at gravitational forces smaller than 500 × *g*, and the generated volume force pushed the biological samples to two incorporated nanofilters. The first set of nanofilters with 600 nm pore size captured large particles while EVs were enriched during the second round of filtration with 20 nm pore size nanofilter, allowing for the elimination of non-vesicular proteins (76). A label-free microfluidic filtration platform to purify EVs from whole blood samples was also developed (95). *In situ* photo patterned porous polymer monoliths [PPM, (96)] were utilized as filter membranes in the proposed microfluidic device. These nanoporous membranes allow the passage of small vesicle-sized particles, while trapping cells and larger debris from blood samples. An electrophoresis cross-flow filtration is also applied in the PPM filter zone to inhibit the clogging of pores with large debris and cells from the bulk stream (95).

### Viscoelastic Flow

Viscoelastic microfluidics is a passive label-free technique for nanoparticle separation that relies on the difference between elastic lift forces imposed on particles with different sizes in a viscoelastic medium (97). Poly(ethylene oxide) (PEO) and poly- (vinylpyrrolidone) (PVP) aqueous solution are two frequently biocompatible synthetic polymers used as viscoelastic media. The inertial forces are extremely small and negligible in these high-viscosity fluids, whereas elastic force is the dominant lateral force exerted on particles. Imbalance in the first (*N*_1_) normal stress difference between the center line and the sidewalls of the microchannel in a non-Newtonian diluted polymer solution results in lateral migration of suspended particles away from the wall to an equilibrium position in the mid-plane. For most of the polymeric solutions, the magnitude of the second (*N*_2_) normal stress difference is small compared to *N*_1_ and does not have considerable impact on dislocating the particles. Liu et al. (77) introduced a size-dependent label-free viscoelastic microfluidic system to extract exosomes from cell culture media or serum with separation purity and recovery rate of more than 90 and 80%, respectively (Figure 3D). A PEO solution was used to enable viscoelastic forces on the EVs, in a controllable manner in the system. The size-dependent lateral displacement of EVs happened through elastic lift force exerted on nanoparticles, where larger nanoparticles align in the center of the microchannel and are collected from the middle outlet, while smaller ones remain near the microchannel sidewalls and are collected from side outlets (77).

## EVs as Biomarkers of Embryo Quality

A large number of studies have characterized and investigated the function of EVs in reproduction, including characterization of EVs recovered from male and female reproductive tract fluids such as the prostate (98), epididymis (98), vagina (99), endometrium (100), follicle (101) and oviduct (102, 103), and their roles in physiologic and pathologic reproductive processes (103–106). However, only a few studies have investigated the role of EVs as a pre-implantation embryo quality marker (9, 107–113). It is important to note that, to date, the only method for investigating mammalian embryo-derived EVs is by using *in vitro* embryo production and analyzing its culture media. This meticulous culture system comes with two major challenges: (i) small sample volume—human embryos are normally cultured individually in microdrops (10–50 μL), which leads to reduced sample size, and low concentrations of embryonic secretions; and (ii) contamination with non-embryonic EVs—the culture media can carry EVs, such as from serum supplementation, that will interfere with the analysis of embryonic-derived EVs. Embryonic EV size, number and cargo (DNA, microRNA and proteins) have been studied as potential biomarkers of embryo quality. It is known that EV secretion and composition are not a random process, and that they change according to external stimuli and active signaling pathways in the cell (114). Therefore, embryonic EVs can be both a mirror of embryo “health” as well as a way for the embryo to control the establishment of a healthy pregnancy with successful embryonic-maternal communication. Embryonic EVs can be studied aiming to understand this embryonic-maternal communication or, as will be discussed here, by directly focusing on the embryo “health status” (developmental competence) as seen by changes in the sizes and numbers of EVs produced, as well as EV cargo composition.

### Size and Number of EVs as Biomarkers of Embryo Quality

**Figure 4:**
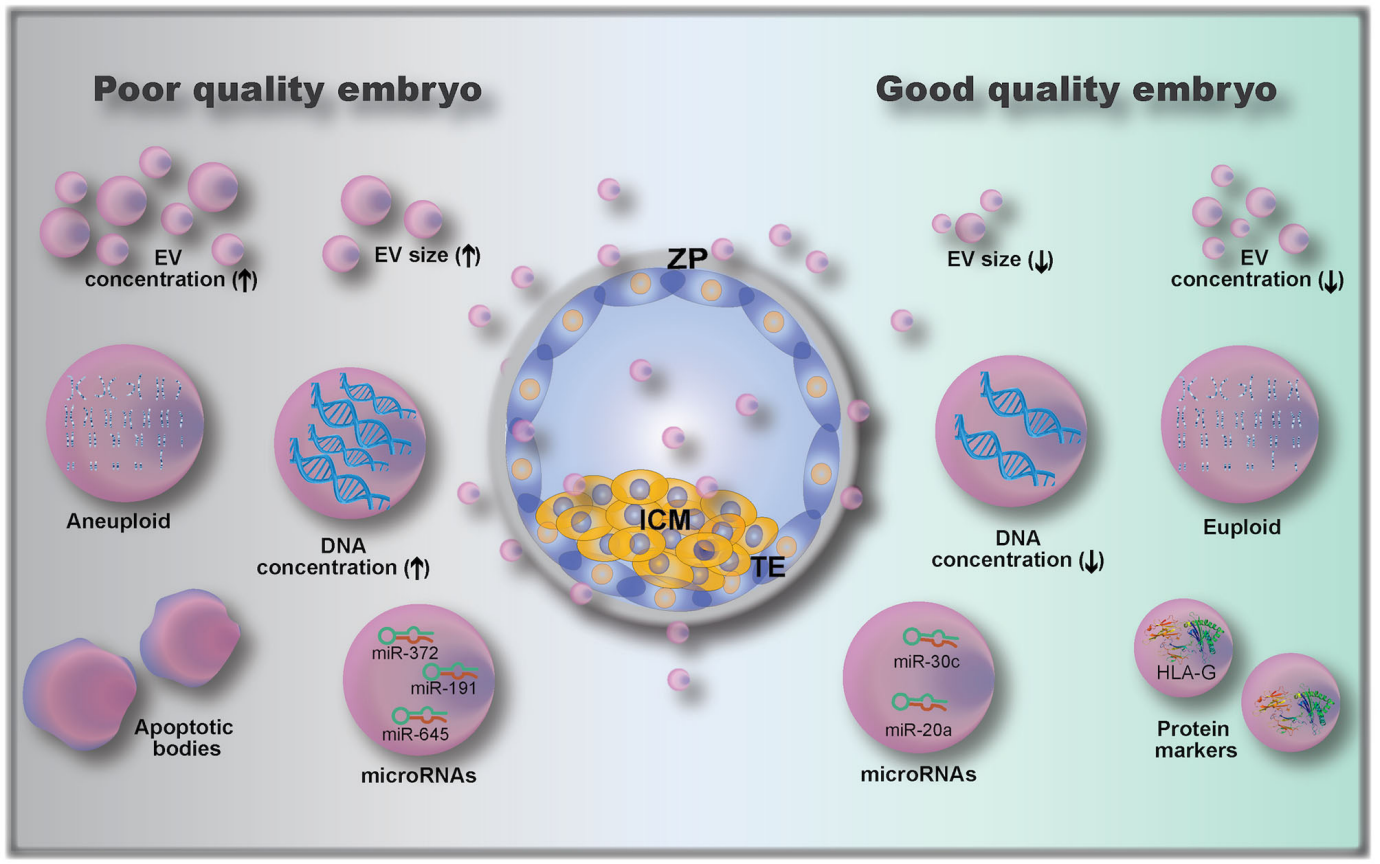
Embryo-derived extracellular vesicles (EVs) and their possible role as biomarkers of embryo quality, including: EV size (110); EV concentration (112); microRNAs (112, 115, 116) and protein cargoes (107, 108); DNA concentration (111); and genetic testing of EVs gDNA (euploid vs. aneuploid) (113). ZP, zona pellucida; ICM, inner cell mass; TE, trophectoderm.

The analysis of embryo-derived EVs in animals and humans is a relatively new field that has not been fully explored, due to the limitations on methods to detect and analyze such low volume and low concentration samples (9). Only a few studies have investigated embryonic EVs and their potential use as embryo quality biomarkers; existing knowledge is summarized in Figure 4. A first attempt to use EVs as biomarkers of embryo competence was made by hypothesizing that cultured human embryos would release EVs and that their size and concentration could reflect embryo quality (110). In this study, the correlation between embryo morphology (assessed by standard morphological criteria) and EV size and concentration was investigated in 239 embryos from 18 women (110). The authors found that increasing EV size was associated with decreasing embryo quality, with bigger vesicles detected in arrested development embryos. Moreover, they also showed that the EV concentration varied with the developmental stage, being higher in day 5 compared to day 3 embryos (110). This was further investigated by Abu-Halima et al. (112) who correlated the number and size distribution of EVs in embryo-spent culture media (9 μL of spent media, collected from embryos cultured individually) with pregnancy rates after transfer. It was observed that the total number of EVs decreased by 1.93-fold in females with positive pregnancy outcomes compared to non-pregnant females (3.8 × 10^9^ vs. 7.35 × 10^9^ particles/mL in pregnant and non-pregnant women, respectively). A variation in EV size distribution was also observed between EVs from embryos that resulted in pregnancies vs. those that did not (112).

### EV Cargoes as Biomarkers of Embryo Quality

In addition to determining EV size and concentration as biomarkers of embryo quality, EV cargo composition of the pre-implantation embryo is also important. The analysis of embryonic EV DNA content by measuring DNA positive EVs using flow cytometry has been described as a non-invasive embryo selection method (111). EVs (isolated from single cultured embryos resulting in either pregnant or non-pregnant outcomes after transfer, 88 patients) labeled with the DNA dye propidium iodide (PI) were analyzed for PI+ intensity by flow cytometry (111). EVs from embryos with successful pregnancy outcomes had lower numbers of PI+ EVs than those from the non-pregnant group. Moreover, the authors determined a cut-off value of 957 PI+ EV count, which corresponded to a 0.9 sensitivity and 0.857 specificity (111). Although the authors proposed a cut-off value based on their data, they suggested that the embryo with the lowest PI+ EV count among all embryos produced from the same mother should be the one selected for transfer (111).

Another recent study has investigated the presence and role of microRNAs (miRNAs) in embryo-derived EVs. miRNAs are small, conserved, single-stranded, non-coding RNA of approximately 22 nucleotides in length (115, 117). They function as molecular switches that bind to complementary sequences of mRNAs, which may then be degraded, downregulated or upregulated (117). miRNAs were already described to be secreted by EVs from embryos of different species, including human, bovine, and porcine (112, 117–120). miRNAs were shown to modulate the embryonic-maternal communication, as seen by modulation of maternal genes following interactions between embryonic miRNAs and uterine cells (118, 121). Analysis of miRNAs released in the embryo culture media (individually cultured embryos) has shown that embryos which established a pregnancy had 103 miRNAs (out of 621 identified miRNAs) differentially expressed when compared to embryos that did not result in a pregnancy (112). Interestingly, another study revealed that miR-20a and miR-30c were present at higher concentrations in culture media (single cultured embryos) from embryos that implanted vs. those that did not implant (115). Rosenbluth et al. (116) demonstrated that miR-191 was highly concentrated in culture media (individually cultured embryos) from aneuploidy embryos, and that miR-191, miR-372, and miR-645 were highly concentrated in culture media from failed IVF embryos. Altogether, these results demonstrate that miRNAs are potential biomarkers of embryo quality, but more research on their role is clearly needed.

Embryonic EVs were also used as a non-invasive method of performing pre-implantation genomic testing (113), as they were shown to contain genomic DNA (gDNA). By using microarray-based comparative genomic hybridization, EV gDNA was evaluated, and a representation of all 23 pairs of chromosomes was present (113). However, when comparing with embryonic biopsy (cell extracted gDNA), the rate of similitude between chromosome abnormalities detected in individually cultured embryos and their respective EVs was 70–80% (113). In addition to gDNA and miRNA analysis, the characterization of embryonic EV proteins can also serve as a quality biomarker. Giacomini et al. (108) have shown that embryonic EVs carry a high amount of HLA-G (histocompatibility antigen, class I, G). Although the authors did not investigate whether HLA-G is differentially expressed between embryos of varying developmental competence, Rizzo et al. showed that HLA-G plays a key role in implantation by controlling trophoblast invasion and maintaining local immunosuppressive state (14). The HLA-G antigen (sHLA-G) has also been shown to play a predictive role in pregnancy outcomes (122), indicating a possibility for quantification of sHLA-G in embryonic EVs as a measure for embryo developmental competence.

### Prospective Use of Microfluidics to Assess EVs as Biomarkers of Embryo Quality

Lab-on-a-chip (LOC) platforms have revolutionized the field of miniature and portable chemical and biomolecular analytical systems and are capable of precise manipulation and rapid detection of a vast number of analytes. By using these LOC systems, conventional macroscale chemical and biological processes have been scaled down in terms of both sample size and device footprint (123). In recent years, significant effort has been made to implement the use of these microfluidic technologies to improve assisted reproductive technologies (ARTs). Microfluidics have already been applied to: sperm capacitation and selection [as reviewed by (124)]; oocyte maturation and selection (125, 126); *in vitro* fertilization and embryo development [reviewed by (127–129)]; ovary-, oviduct- and testis-on-a-chip development (130–133); full menstrual cycle-on-a-chip development (134); and gametes and embryo cryopreservation (135, 136). Although the use of microfluidic technologies for ARTs and EV isolation/characterization has grown in the past years, the use of microfluidics specifically for embryonic EV isolation has yet to be established. Among the benefits of using microfluidics for EV isolation are: overcoming the challenge of low concentration and small sample volume when isolating embryonic EVs; providing a user-friendly standardized and automated method of EVs isolation and characterization in different laboratories; rapid processing of samples; as well as being low cost, as seen by reduced use of reagents (microscale) and elimination of expensive equipment. In addition to microfluidics overcoming the challenges of embryonic EV isolation from low concentrated samples, this technology can also be used to perform on-chip characterization of EVs, allowing non-invasive and time-dependent analyses to be performed. A summary of advantages and challenges of microfluidics over conventional methods to isolate EVs is presented in Table 3. Among the embryonic EV characteristics that can be used as embryo quality biomarkers, five of them have the potential to be automated in a microfluidics platform for quick characterization: (1) size of EVs; (2) number of EVs; (3) EV protein markers; (4) EV DNA concentration; and (5) EV microRNA markers. For all models, at least two steps must be performed in the device: (1) isolation and (2) characterization.

**Table 3:** Advantages and challenges of conventional and microfluidics methods of EVs isolation.

	**Conventional methods**	**Microfluidics methods**
Advantages	Capable in scaling up a single experiment Well-established protocols Available assays and standardization	Small device footprint Low contamination risk Low sample consumption Easy in integration of multi-step protocols Easily disposable Rapid and easy fluid manipulation Real time process monitoring Flexible in changing the protocols or experimental setup Low shear force on biological samples
Challenges	Low recovery rate Fixed device design and architecture High volume consumption Limited to end-point analysis Labor intensive and trained technicians for running experimental tests Less control over the process High maintenance cost	Complex operational control and manufacturing Requires multiple components such as pump, valve, tubing, and connectors for operation Standardization and industrialization Sensitive to operational conditions Difficult for mass production

### On-Chip Embryonic EV Isolation

As discussed above, both traditional and microfluidic EV isolation methods have specific advantages and pitfalls. Embryonic EV isolation methods should, ideally, allow the use of small volume samples, have high yield (working with low EV concentration samples), and detect a wide range of EV sizes. Therefore, microfluidics appears as a promising technique to isolate embryonic EVs, since it allows high yield and purity of EV isolation from low sample volumes. Specifically, on-chip filtration and acoustofluidic separation appear to be the most promising methods, since they are label-free methods which allow the isolation of a higher EV size-range (20–6,000 nm) with high recovery, high specificity and short time (<30 min) (71, 74–76).

### On-Chip Embryonic EV Characterization

#### Micromixers for On-Chip EVs Labeling

A micromixer is a device based on mechanical microparts used to mix fluids. Sample mixing in microfluidic devices is achieved by external turbulences and/or special microstructures inside the microchannels to obtain a larger surface-to-volume ratio and increasing heat and mass transfer efficiency (137). Micromixers are classified into two categories: passive and active mixers. Active micromixers can be driven by pressure, sound, electrical, thermal and magnetic fields, while passive micromixers, can be developed by unbalanced collisions, two-dimensional obstacles, three-dimensional lamination, spiral and convergence-divergence structures [reviewed by (137)]. Most of the EVs characterization methods we will discuss here require the micromixing technologies to boost the on-chip biochemical detection assays, such as labeling EVs with commonly used lipophilic fluorescence (PKH26, DiO, DiA, DiI, and DiR), and DNA (propidium iodide) dyes. The integration of micromixers with biochemical sensors is a necessary and promising development.

#### Nano Flow Cytometer and On-Chip Microscopy to Characterize EVs Concentration, Size and DNA Cargo

A nano flow cytometer device for fluorescence-based detection and characterization of small lipid vesicles was created by Friedrich et al. and accurately counted the number of lipid vesicles down to a concentration of 170 fM using only 20 μL of sample volume (138). This device works by individually visualizing vesicles by fluorescence microscopy during their passage through hundreds of parallel nanochannels in a pressure-driven flow (138). Moreover, it can be imaged under a standard epi-fluorescence microscope, which provides an easy method of measurement and detection (138). This nano flow cytometer can also resolve the size-distribution of the lipid vesicles, based on the vesicle's fluoresce intensity (138). To make this device portable and ready to use without the need of a fluorescence microscope, on-chip fluorescence analysis can be used. Several studies have demonstrated the creation of different kinds of on-chip fluorescence analyses: contact fluorescence microscopy (139), random microlens diffuser (140), tapered fiber-optic faceplate (141), and spectrally filtered passive Si photodiode array (142). Most promisingly, on-chip light sheet illumination accurately tracked fluorescence labeled single EVs, characterizing their size and concentration in cell culture medium and interstitial fluid collected from primary human breast tumors (143). The combination of these technologies allows the characterization of EV size and concentration, when labeled with lipophilic dyes, and EV DNA cargo if, instead of labeling with lipophilic dyes, EVs are labeled with the DNA marker PI.

#### On-Chip ELISA and Western Blot to Characterize EV Protein Cargo

An enzyme-linked immunosorbent assay (ELISA) LOC was designed and developed for field-use (123). This device had the flow operated by three micropumps, which replicated the steps of a normal ELISA: Analyte perfusion and incubation for binding to the primary antibody (coated in a detection chamber); secondary antibody incubation; colorimetric substrate incubation; and wash of unbound antibodies/substrates (123). This ELISA-on-a-chip is attached to an universal serial bus (USB)-interfaced mobile platform to control, execute, and read microfluidic-based immunoassays (123). This smart-phone interfaced ELISA-on-a-chip was used to detect the presence and concentrations of BDE-47 (2,20,4,40-tetrabromodiphenyl ether), an environmental contaminant found in our food supply with adverse health impact (123). Interestingly for embryonic EV analysis, this ELISA-on-a-chip requires low sample volume (2 μL) and has high sensitivity (concentration range of 10^−3^-10^4^ μg/L).

Western blot (WB) is an essential analytical tool, benefiting clinical diagnostics and fundamental questions in the life sciences (144). On-chip WB was already described and can also be used as a tool for EV protein detection (144). Specifically, a μWB was created by combining isotachophoretic sample stacking during sample injection, weight-based separation of denatured protein analytes through SDS-PAGE, and *in situ* immunoblotting with fluorescently labeled primary and secondary antibodies (144). This μWB advances four key aspects of analytical performance: exceptional protein blotting efficiency with near complete analyte capture, accelerated run times (10–60 min), small device footprint (800-fold smaller device area compared with conventional gel lane), and outstanding reagent economy, with a 10^3^-fold reduction in antibody and buffer requirements over conventional WB. Important to embryonic EV protein measurements, the μWB requires low starting sample concentration (low picomolar), and low starting sample total mass and volume (144).

#### On-Chip EV miRNAs Detection

A simple and rapid PCR-free microfluidics device to detect EV miRNAs was developed by integrating surface acoustic wave (SAW) to perform EV lysis, concentration and sensing in a microfluidic device. This device incorporates an electrokinetic membrane sensor that is based on non-equilibrium ionic currents (ion exchange membrane—IEM) (145). Complimentary target miRNAs probes are attached to the IEM, to allow miRNAs hybridization and detection. For detection of target miRNA, the depletion side of an anion-exchange membrane (AEM) is used where the hybridization of target miRNA with oligoprobes attached to the AEM reduces the ion depletion action, resulting in shifted over-limiting current in the current–voltage curve. This large voltage shift, due to gating of the depletion ion current by the hybridized miRNAs is much larger than voltage signals from electrochemical sensors and offers sensitive quantification of hybridized miRNAs (145). Differently from conventional RT-qPCR methods, this technology does not require RNA purification, reverse transcription, or amplification (145). Crucial for embryonic EV characterization, this device is capable of absolute quantification (<10% uncertainty) of EV miRNAs with 1 pM detection sensitivity and requires ~20 μL of sample (145). Moreover, the assay is completed in only 30 min as opposed to the 13 h time period required for conventional RT-qPCR techniques (145).

## Conclusions

The selection of the most fit embryo for transfer and successful pregnancy remains an under-developed field, with many future directions to pursue. The study of EVs as biomarkers has exponentially grown in the past years, specifically in the immunology and cancer areas. Additionally, as reviewed here, EVs hold promising perspectives for use as pre-implantation embryo quality biomarkers. However, their widespread use for embryo quality has been hindered by low sample volume and EV concentration, which limits single embryo EV isolation and characterization using conventional methods. The optimization of EV isolation and characterization using microfluidic technologies is encouraging and has yet to be included in the reproduction field. Although microfluidic technologies can improve our ability to study EVs in a faster, more sensitive manner, the lack of a gold-standard embryo quality marker limits its routine use in IVF clinics. Consequently, the immediate application for non-invasive microfluidic EV analyses reviewed here are more toward selecting the most fit embryo among a pool of embryos from the same donor rather than creating devices to evaluate embryo quality based on biomarker thresholds. Yet, microfluidics is a promising technology that should be explored.

## Author Contributions

All authors contributed to the article, reviewed, and approved the submitted version.

## Conflict of Interest

The authors declare that the research was conducted in the absence of any commercial or financial relationships that could be construed as a potential conflict of interest.
